# Kinesiophobia in patients with coronary heart disease: a Rodgers’ evolutionary concept analysis

**DOI:** 10.3389/fpsyg.2025.1499962

**Published:** 2025-03-26

**Authors:** Mei-Jun Zhang, Si Liu, Xiao-Yun Xiong, Meng-Die Liu, Qin Xiang

**Affiliations:** ^1^Department of Nursing, The Second Affiliated Hospital of Nanchang University, Jiangxi Medical College, Nanchang University, Nanchang, Jiangxi, China; ^2^School of Nursing, Jiangxi Medical College, Nanchang University, Nanchang, Jiangxi, China

**Keywords:** fear of movement, ischemic heart disease, chronic diseases, physical exercise, physical activity, cardiac rehabilitation

## Abstract

**Introduction:**

Research on kinesiophobia in coronary heart disease is increasing, but existing studies primarily adopt concepts from the chronic pain domain, neglecting the specific characteristics of coronary heart disease patients. This gap limits effective identification and management.

**Objective:**

This study aims to clarify the concept of kinesiophobia in coronary heart disease using Rodgers’ evolutionary concept analysis.

**Methodology:**

Rodgers’ evolutionary concept analysis method was applied to review the literature. A comprehensive search was conducted in PubMed, Web of Science, PsycINFO, CINAHL, Cochrane Library, Embase, Scopus, ProQuest, OVID, CNKI, Wanfang Data, CBM, and VIP Database (up to June 30, 2024). Inclusion criteria: Studies on coronary heart disease patients, addressing the concept’s attributes, antecedents, and consequences, published in English or Chinese. Exclusion criteria: Unavailable full text, gray literature, non-peer-reviewed texts, and study protocols.

**Results:**

A total of 31 articles were included. The attributes of kinesiophobia in coronary heart disease patients were identified as self-symptomatic distress, complex emotional responses, subjective avoidance behavior with personality tendencies, and misperceptions with negative reactions. Antecedents included sociodemographic, disease-related, and psychological factors. Consequences encompassed reduced participation in cardiac rehabilitation, decreased functional capacity, increased major adverse cardiac events, and lower quality of life.

**Conclusion:**

In this study, we found that kinesiophobia in patients with coronary heart disease is a subjective avoidance behavior that includes both “fear of pain or weakness” and “fear of cardiac events” based on personality tendencies, driven by complex emotional responses and misperceptions based on their own symptomatic disturbances, and presents an excessive and irrational fear of movement. This analysis highlights the need for early identification and multidisciplinary interventions tailored to this population. It also provides a foundation for developing more specific and objective assessment tools.

## 1 Introduction

Coronary heart disease (CHD), caused by coronary artery atherosclerosis leading to myocardial ischemia, hypoxia, or necrosis, is the leading cause of death globally (Timmis et al., 2020). Patients often face recurrent episodes, frequent hospitalizations, and repeated interventions like coronary angiograms and revascularizations. CHD poses a serious threat to life, health, and imposes a heavy economic burden on individuals and healthcare systems worldwide (Roth et al., 2020; Timmis et al., 2020; Virani et al., 2021). Currently, 423 million people suffer from CHD, accounting for 31% of all deaths annually (Lum et al., 2020). The World Heart Association projects global cardiovascular costs to rise from $863 billion in 2010 to $1,044 billion by 2030 (Virani et al., 2021).

Cardiac rehabilitation (CR) plays a crucial role in secondary prevention for CHD, helping to stabilize, slow, or even reverse atherosclerosis progression. It reduces overall mortality by 13 to 24% and readmission rates by 31% within a year (Resurrección et al., 2019). This comprehensive program spans the acute, stabilized, and lifelong phases of CHD and includes medication, exercise, nutrition, psychosocial support, risk factor management, education, and lifestyle guidance (Knuuti et al., 2020). Exercise rehabilitation, the cornerstone of cardiac rehabilitation, improves myocardial circulation, lowers mortality and readmission rates, reduces functional impairments, and enhances quality of life (Fu et al., 2019; Dibben et al., 2021). However, global participation in exercise rehabilitation remains low at 20–50%, with even lower rates in developing countries (Bäck et al., 2016). A major barrier is kinesiophobia, which affects participation and adherence, negatively impacting patients’ quality of life (Bäck et al., 2016; Knapik et al., 2019; Baykal Sahin et al., 2021; Yang et al., 2023a; Yang et al., 2023d). Studies have shown that 25.4% of patients with CHD have kinesiophobia throughout the course of the disease (Keessen et al., 2020a). Therefore, the identification and intervention of kinesiophobia in patients with CHD is an important topic and challenge.

The term “fear of movement” originated in chronic pain research and refers to avoidance behaviors driven by fear of pain (Lethem et al., 1983). It has since been studied in post-surgery (Masuy et al., 2022), stroke (Wasiuk-Zowada et al., 2021), and heart failure patients (Sentandreu-Mañó et al., 2024). While kinesiophobia is common across patient groups, its triggers and nature vary, making generalization inappropriate. For example, chronic pain patients fear pain-related movements due to catastrophic perceptions of pain (Vlaeyen and Linton, 2012). In contrast, angina in CHD patients is often sudden, brief, and triggered by factors like cold, diet, emotions, or exercise. This pain may signal myocardial ischemia, raising serious concerns about vital organs and death (Bäck et al., 2018). Additionally, Bäck et al. (2012) patients diagnosed with CHD may experience existential fears related to exertion and adverse events due to the specificity of their symptoms. These fears may include a fear of death, concerns about stent dislodgement or displacement during exercise, and anxiety about experiencing another cardiac event. Although kinesiophobia has evolved in coronary heart disease (CHD) patients, its unique characteristics remain underexplored, and despite the focus on exercise interventions, the lack of a conceptual analysis limits the development of targeted interventions and assessment tools, making a clear understanding essential for improving rehabilitation outcomes. Conceptual analysis is essential to address this gap and guide the creation of more precise and effective strategies for managing kinesiophobia in this patient group.

Concept analysis is an effective method for clarifying ambiguous terms widely used across disciplines. Rodgers’ Evolutionary Concept Analysis emphasizes that concepts are dynamic and evolve over time (Rodgers, 1989). To refine their current usage, this approach redefines context, attributes, surrogate and related terms, antecedents, model cases, and consequences, providing a structured foundation for research and practice (Tofthagen and Fagerstrøm, 2010). Unlike traditional methods, evolutionary concept analysis traces a concept’s historical development, examines its key components, explores diverse interpretations, and analyzes its real-world applications (Rodgers et al., 2018). By emphasizing conceptual fluidity and multidimensionality, this method is particularly suitable for studying evolving, cross-disciplinary concepts, offering valuable insights into their theoretical development and practical implications.

This study aims to analyze kinesiophobia in CHD patients using Rodgers’ evolutionary approach, hypothesizing that it is a complex phenomenon influenced by cognitive distortions, emotional responses, and individual personality traits, rather than merely a psychological condition. By examining its core characteristics, this research seeks to provide a clearer theoretical framework to support early identification and the development of effective interventions for kinesiophobia in CHD patients.

## 2 Methods

### 2.1 Concept analysis method

Rodgers’ evolutionary conceptual analysis was employed to clarify the concept of kinesiophobia in CHD patients. This method, based on Walker and Avant’s classical approach, focuses on clarifying vague or abstract concepts by analyzing their usage within a discipline, emphasizing changes over time and across contexts (Tofthagen and Fagerstrøm, 2010). Rodgers’ method involves six steps, as outlined in Table 1.

**Table 1:** Steps for Rodgers’ evolutionary concept analysis.

Step	Description
1	Identify the concept of interest and related expression
2	Identify and select appropriate fields for data collection
3	Identify attributes and analyze antecedents and consequences
4	Analyze and summarize data about conceptual features
5	Identify examples of the concept
6	Identify implications for further development of the concept

### 2.2 Data sources

Studies on kinesiophobia in patients with CHD were searched in PubMed, Web of Science, PsycINFO, CINAHL, the Cochrane Library, Embase, Scopus, ProQuest, OVID, CNKI, Wanfang Data, CBM, and VIP Database from library build to June 30, 2024. Additional articles were manually retrieved from reference lists. The search terms used were a combination of MeSH terms and free-text words, categorized as follows:

**Table:** 

Concept	Search terms
Population	Coronary disease; coronary heart disease; coronary atherosclerotic heart disease; coronary atherosclerosis; atherosclerotic cardiovascular disease; ischemic heart disease; myocardial ischemia; acute coronary syndrome; myocardial infarction; angina pectoris; percutaneous coronary intervention; PCI; percutaneous transluminal coronary angioplasty; coronary artery bypass
Kinesiophobia-related	Kinesiophobia; pain-related activity avoidance; movement phobia; fear of movement; kinesophobia; kinetophobia; phobia, movement; fear of reinjury; motion phobia; fear of activity; fear avoidance

Inclusion criteria: (1) studies on CHD patients; (2) research addressing the concept’s attributes, evolution, antecedents, and consequences; (3) published in English or Chinese. Exclusion criteria: (1) unavailable full text; (2) gray literature, non-peer-reviewed texts, letters, and study protocols. Detailed search strategies are provided in Supplementary material 1.

### 2.3 Flow of included articles

**Figure 1:**
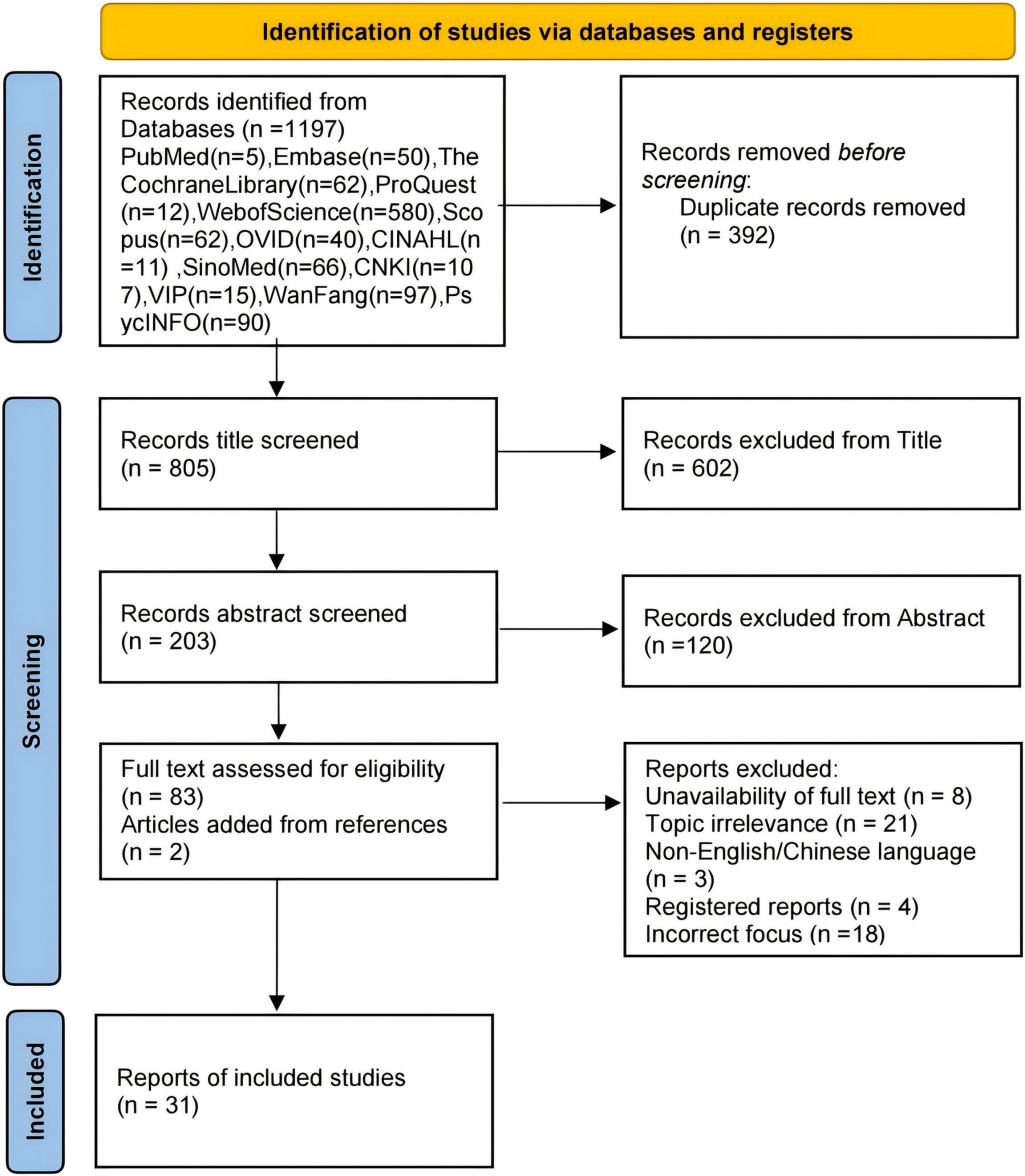
Flowchart of the study selection process of the concept analysis.

In this study, two researchers independently screened the literature, with a third resolving any disagreements. After reviewing the selected studies, the researchers extracted information for conceptual analysis. Initially, 1,197 studies were retrieved, and after removing duplicates, 805 remained. Following title and abstract screening, 722 were excluded, leaving 83 for full eligibility assessment. We excluded 54 studies due to unavailability of full text (*n* = 8), topic irrelevance (*n* = 21), non-English/Chinese language (*n* = 3), registered reports (*n* = 4), and incorrect focus (*n* = 18). Two additional articles were sourced from reference lists, resulting in 31 articles for conceptual analysis. A detailed flow chart is shown in Figure 1, with exclusion reasons in Supplementary material 2.

## 3 Results

In this section, we present the results of the concept analysis, organized into several key components: study characteristics, the evolving definition and use of the concept, surrogate terms and related concepts, attributes, antecedents, consequences, model case, and the final definition of kinesiophobia in patients with CHD. Additionally, we discuss the measurement tool used to assess this concept.

### 3.1 Study characteristics

Based on the inclusion and exclusion criteria, 31 papers were finally included for conceptual analysis, 25 in English and 6 in Chinese. Most used cross-sectional and qualitative research designs. Study details are provided in Table 2.

**Table 2:** Characteristics of the included studies.

Author (Year)	Country	Attributes	Antecedents	Consequences
Zhang et al. (2023)	China	Symptom disturbance (fatigue, shortness of breath, chest tightness); fear of cardiac events	Self-efficacy	Self-management behavior related to physical activity
Zhu et al. (2024)	China	Misperceptions; complex emotional responses (worry, fear, anxiety); Subjective avoidance behavior (the traditional concept of bed rest);	Education level; clinical classification of CHD; exercise habits, social support	QoL
Yakut Ozdemir et al. (2023)	Turkey	Self-symptomatic distress (dyspnea); information support from healthcare professionals	Complication; exercise habits	CR participation; QoL
Çakal et al. (2022)	Turkey	Symptomatic limitation (shortness of breath, disease duration, a major surgical approach, angina pectoris); misconceptions	Anxiety	QoL
Wang et al. (2023)	China	Inability to accurately differentiate between the effects of exercise and disease on physical status; avoidance behavior (the traditional concept of bed rest)	Age; education level; complication; anxiety	QoL
Bäck et al. (2018)	Sweden	Complex emotional responses (anxiety, depression, fear); subjective avoidance behavior with personality tendencies	Gender; anxiety; depression	Attendance and adherence to exercise-based CR
Liu et al. (2024)	China	Concerns about symptoms (angina, heart failure); misperceptions and negative reactions	Education level; pain scores; cardiac exercise self-efficacy; social support	QoL; functional capacity
Shen et al. (2023)	China	Self-symptomatic distress (angina)	Personal monthly income; NYHA classification; pain intensity and pain resilience	Functional capacity
Kluszczyńska et al. (2021)	Poland	A manifestation of personality predisposition to physical activity; irrational fear; related to psychosomatic symptoms (pain, fatigue); complex emotional responses (exhaustion, anxiety, fear of ridicule)	Anxiety; gender; age; education level	QoL; functional capacity
Piao et al. (2022)	China	Fear of angina pectoris; complex emotional responses (anxiety, fear); healthcare professionals’ views or recommendations	Self-efficacy	Physical activity
Li L. et al. (2024)	China	Fear of cardiac events	Self-efficacy; fatigue	Functional capacity
Ratnoo et al. (2023)	USA	Negative beliefs and attitudes; fear of getting injured; psychological adaptations	Depression; anxiety	Impaired shoulder functions; impaired physical performance; self-reported disability; MACE; mortality rate
Bäck et al. (2013)	Sweden	Insufficient awareness; Subjective avoidance behavior; healthcare provider awareness and perceptions; concerns about self-symptoms	Complication; anxiety	CR participation; functional capacity; QoL
Shea et al. (2023)	USA	Motion safety Perception	Self-efficacy	Mental health; physical health; QoL; exercise tolerance
Özden et al. (2023)	Turkey	Complex emotional responses (depression, pain catastrophizing); subjective avoidance behavior with personality tendencies	Depression; anxiety	Physical activity
Knapik et al. (2019)	Poland	Fear of pain or weakness; fear of cardiac events; emotional disorder; associated with an existing physical illness	Age; personal monthly income	Functional capacity
Khushnood et al. (2023)	Pakistan	Fear of weakness; irrational fear; fear of cardiac events	Self-efficacy	Functional capacity; QoL
Demirsoy et al. (2024)	Turkey	Misperceptions and negative reactions	Age; complication; pain	6MWT; QoL
Demirci et al. (2023)	Turkey	Self-symptomatic distress (pain, palpitations, dyspnea, weakness); fear of cardiac events; complex emotional responses (stress, anxiety, fear); subjective avoidance behavior	Anxiety	QoL
Li Q. et al. (2024)	China	Worry about heart problems; fear of re-admission; related to physiological and psychological factors; negative emotion (anxiety, depression, pain catastrophizing); misperceptions;	Self-efficacy, psychogenic anxiety; social support	CR participation and adherence; functional capacity
Brunetti et al. (2017)	Italy	Negative emotion (high perceived pressure)	Age; education level; anxiety; complication	Self-image; self-confidence; QoL
Baykal Sahin et al. (2021)	Turkey	Poor self-assessment of health	Disease duration; clinical classification of coronary heart disease	CR participation and adherence; functional capacity; MACE; mortality rate; QoL
Bäck et al. (2020)	Sweden	Complex emotional responses (ambivalence, hypervigilance, catastrophizing thoughts); fear of cardiac events; subjective avoidance behavior with personality tendencies; ambiguous information; Tend to rest; self-symptomatic distress (increased heart rate, shortness of breath, pain)	Self-efficacy	CR participation and adherence; Disability
Guo et al. (2022)	China	Self-symptomatic distress (dizziness, chest pain, chest tightness); fear of stent detachment or displacement after surgery; irrational fear; weakness; emotionally driven withdrawal behavior	Anxiety; depression	CR participation and adherence; QoL
Wang Z. et al. (2022)	China	Fear of cardiac events; fear of death; sports adaptation psychology	Age; psychogenic anxiety; depression	Disability; readmission rates; MACE; mortality rate; QoL
Bäck et al. (2017)	Sweden	Ear of experiencing another cardiac event; fear of death; anxiety; looking for support from a physiotherapist	Self-efficacy; age	Functional capacity; QoL
Xiao et al. (2024)	China	Consider increased heart rate and shortness of breath during activity as red flags; hypervigilance and sensitivity to one’s own state; subjective avoidance behavior	Age; complication; monthly personal income; BMI; NYHA classification; exercise habits	Functional capacity
Wang et al. (2022a)	China	Fear of cardiac events; decreases with time	Age; self-efficacy; exercise habits; social support; depression	QoL
Song et al. (2022)	China	Subjective avoidance behavior with personality tendencies; negative emotion (pain catastrophizing); fear of cardiac events	Pain; fatigue; anxiety	CR participation and adherence; functional capacity; QoL
Wang et al. (2022b)	China	Inadequate knowledge of exercise rehabilitation; fear of cardiac events; avoidance of stressful events; negative emotions; Hypervigilance to one’s own state	Negative emotions	Exercise self-efficacy; CR participation and adherence; QoL
Ren et al. (2022)	China	The traditional concept of bed rest; insufficient awareness; the influence of healthcare professionals’ attitudes	Self-efficacy	CR participation and adherence

CHD, coronary heart disease; CR, cardiac rehabilitation; QoL, quality of life; MACE, major adverse cardiovascular events; NYHA classification, New York Heart Association functional classification; BMI, body mass index; 6MWT, six-minute walking distance.

### 3.2 Evolving definition and use of the concept

Since “kinesiophobia” is not defined in dictionaries, we analyzed its components separately: “kinesis” and “phobia.” In the Oxford Dictionary, “kinesis” means “movement,” referring to “an act of moving the body or part of the body,” while “phobia” is defined as “a strong unreasonable fear of something.” PubMed defines “kinesiophobia” as an anxiety disorder of persistent and irrational fear of movement following an injury. It is related to perceived disability due to injury and catastrophizing in fear of (anticipated) pain and possibility of reinjury.

Lethem et al. (1983) developed the “fear-avoidance” model for pain patients, centering on the fear of pain and distinguishing between “confrontation” and “avoidance” responses. Confrontation involves viewing pain as a temporary nuisance and continuing physical and social activities, while avoidance exaggerates pain, reducing or ceasing movement and leading to severe disability. The model suggests that varying perceptions of negative stimuli can lead to different outcomes. Kori (1990) introduced “kinesiophobia” based on this model to describe fear of movement due to lower back pain, defining it as excessive and irrational fear of movement linked to pain and vulnerability to re-injury. Vlaeyen et al. (1995) expanded the model into a cognitive-behavioral framework, where misinterpretation of exercise or re-injury leads to avoidance behaviors and heightened focus on bodily sensations, worsening the physical and psychological impact of illness. In 2012, Swedish scholar Bäck et al. (2012) first used the Tampa Scale for Kinesiophobia Heart to measure kinesiophobia in CHD patients, and found that 20% of CHD patients suffered from kinesiophobia, which also was the first application of this concept in the field of cardiovascular disease. In this study, the authors followed the concepts proposed in the field of chronic pain. However, it was noted that kinesiophobia in patients with CHD has its own symptomatic peculiarities compared with patients with pain, and that the nature of the perceived threat may vary between different patient groups. Future research is needed to develop a more consistent definition of kinesiophobia in patients with CHD. However, subsequent research has continued using concepts from chronic pain without adapting them for CHD (Bäck et al., 2013; Gołba et al., 2018). Therefore, a consistent definition of kinesiophobia in patients with CHD is necessary.

### 3.3 Surrogate terms and related concepts

Surrogate terms are specific words or phrases used to describe a concept (Rodgers, 1989). In medical literature, “fear of movement” and “kinesiophobia” are often used interchangeable (Keessen et al., 2020a; Keessen et al., 2020b; Çakal et al., 2022). Related concepts share similarities with the main concept but differ in characteristics (Rodgers, 1989). For this study, “exercise anxiety” is the relevant term (Çakal et al., 2022; Liu et al., 2024). Researchers suggest that patients who have experienced cardiac events may exhibit anxiety and fear related to physical activity, which is viewed as part of generalized anxiety disorder and associated with preventive behaviors (Vlaeyen et al., 2012; Liu et al., 2024).

### 3.4 Attributes

Attributes are the defining characteristics of a concept that help identify its presence in a given situation. Identifying these key attributes is essential to distinguish the concept from others (Rodgers and Knafl, 1993). The following attributes of kinesiophobia in CHD patients were summarized from the analysis of the included studies.

#### 3.4.1 Self-symptomatic distress

Kinesiophobia in different patients is highly heterogeneous due to multiple influences and the complex and diverse characteristics of individuals (Bäck et al., 2013). Patients with CHD who experience a cardiac event can be profoundly disturbed by their own symptoms during exercise (Bäck et al., 2013; Shen et al., 2023; Yakut Ozdemir et al., 2023; Liu et al., 2024). The cardiac-related sensations that occur during physical activity, such as chest tightness and shortness of breath, are similar to the symptoms experienced during a heart attack, leading to a sense of impending doom (Bäck et al., 2020; Çakal et al., 2022; Zhang et al., 2023). This makes it impossible for patients to accurately differentiate between the effects of exercise and illness on their physical condition (Yakut Ozdemir et al., 2023; Zhang et al., 2023). As a result, patients may become increasingly concerned about the potential consequences of physical activity, fearing that exercise could exacerbate their existing cardiac or physical condition, leading to complications, rehospitalization, or other adverse events (Kluszczyńska et al., 2021; Liu et al., 2024). This anxiety manifests as a fear of uncertainty regarding their future physical activity (Guo et al., 2022). This is also confirmed by the study of Çakal et al. (2022). Furthermore, it is worth noting that patients with CHD can exhibit a double fear of movement due to the specificity of their symptoms. The first layer involves a fear of pain or weakness, where patients worry that physical activity might exacerbate the pain in the heart region, prolong its duration, or cause pain and fatigue in other areas (Shen et al., 2023; Wang et al., 2023). The second layer is a fear of cardiac events, where patients are concerned that exercise might cause the dislodgement or displacement of stents, lead to another cardiac event, or even result in death (Bäck et al., 2013; Shea et al., 2023). Among these, the fear of experiencing another cardiac event tends to be more pronounced in patients with CHD (Bäck et al., 2017). This is consistent with the findings of Shen et al. (2023). These intertwined fears intensify the patients’ negative beliefs and attitudes toward physical activity (Xiao et al., 2024).

#### 3.4.2 Complex emotional responses

Kinesiophobia encompasses not only a simple fear of physical activity, but also a complex, multifactorial mindset that stems from a belief in vulnerability (Kluszczyńska et al., 2021). Kluszczyńska et al. (2021) noted that past experiences and current physical conditions may lead patients to worry about the possibility of re-injury. This process involves a complex set of emotional responses including worry, fear, anxiety, depression, frustration, catastrophizing of pain, feelings of inadequacy and embarrassment (Bäck et al., 2017; Knapik et al., 2019; Kluszczyńska et al., 2021; Özden et al., 2023). This complex experience of negative emotions prompts patients to over-amplify unpleasant sensations such as pain, chest tightness, shortness of breath and dyspnea, leading to heightened sensitivity to physical activity (Bäck et al., 2018; Knapik et al., 2019; Kluszczyńska et al., 2021; Piao et al., 2022). Consequently, they perceive physical activity as a potential trigger for cardiac events, ultimately resulting in a harmful cycle of “kinesiophobia – reduced physical activity – decreased exercise capacity – kinesiophobia” (Demirsoy et al., 2024). The study by Liu et al. (2024) also emphasized that negative emotional experiences can have a significant impact on patients’ perception of movement as well as motor behavior. A series of psychological issues can lead to negative outcomes, such as reduced adherence to CR, diminished physical activity capacity, increased readmission rates, and a decline in quality of life (Guo et al., 2022; Demirci et al., 2023; Demirsoy et al., 2024). Moreover, these effects are persistent and may even outweigh the direct physiologic harm caused by the cardiac event itself (Özden et al., 2023). Therefore, improving individual emotional responses is crucial in preventing kinesiophobia in patients with CHD.

#### 3.4.3 Subjective avoidance behavior with personality tendencies

In the field of psychology, evidence indicates that temperament and personality traits significantly influence individual behavior (Wang et al., 2022b). Bäck et al. (2018) found that for patients with kinesiophobia, they often tend to be pessimistic about future problems and their personalities are characterized by active avoidance of potential harm. With limited self-awareness, patients tend to judge the intensity and type of exercise their bodies can tolerate based on their previous exercise experience and their own symptoms (Bäck et al., 2013; Bäck et al., 2018; Demirci et al., 2023). The study by Zhu et al. (2024) also showed that personality affects patients’ self-assessment of exercise. In addition, studies have shown that most patients are heavily influenced by the traditional belief that the disease should be controlled by rest and bed confinement (Bäck et al., 2020; Wang et al., 2023). Most of them have a poor level of self-assessment and are therefore hypervigilant about exercise, thus actively avoiding behaviors that may cause injury (Knapik et al., 2019; Ren et al., 2022; Xiao et al., 2024). Thus, kinesiophobia is a manifestation of a personality predisposition toward physical activity (Kluszczyńska et al., 2021).

#### 3.4.4 Misperceptions and negative reactions

Exercise after a cardiac event is a long-term process that requires both internal and external support (Piao et al., 2022; Yakut Ozdemir et al., 2023). Advice or perceptions from healthcare professionals can influence patients’ perceptions of the safety of exercise (Ratnoo et al., 2023). When an individual is in a disease state or when his or her health is threatened, it can aggravate the patient’s false perception of the disease, which directly or indirectly affects the patient’s prognosis, quality of life, and even the recovery of social function (Song et al., 2022; Wang et al., 2022b). During the acute phase, healthcare professionals emphasize the importance of absolute bed rest, reinforcing patients’ concerns and fears about their health. However, even when myocardial blood flow has been restored, they are still in a state of panic and tension, fearing that activity will lead to disease recurrence or deterioration (Piao et al., 2022; Yakut Ozdemir et al., 2023). The reason for this may be that the patient lacks proper knowledge about the disease as well as about exercise. The study by Zhu et al. (2024) also emphasized that if information about physical activity and exercise is unclear, it can increase patients’ kinesiophobia. Bäck et al. (2013) noted that patients’ perceptions of illness influence their coping strategies. The higher the level of negative cognition, the greater the fear of physical activity or exercise-induced adverse cardiac events such as angina, myocardial infarction, and readmission (Bäck et al., 2013; Ren et al., 2022; Wang et al., 2023). As irrational fears increase, the severity of kinesiophobia also intensifies, leading to irrational avoidance of physical activities, which ultimately affects the patient’s quality of life and recovery process.

### 3.5 Antecedents

Antecedents are events or phenomena that precede a concept (Rodgers and Knafl, 1993). In this study, antecedents of kinesiophobia in CHD patients were categorized into three areas: sociodemographic factors, disease factors, and psychological factors.

#### 3.5.1 Sociodemographic factors

The main sociodemographic factors influencing kinesiophobia in CHD patients include age, gender, social support, education level, exercise habits, and personal monthly income. Studies show that as age increases, patients experience a decline in physical function and activity capacity, often with comorbidities, which increases their fear of falling during exercise and leads to higher levels of kinesiophobia (Bäck et al., 2017; Wang Z. et al., 2022; Wang et al., 2023). Kluszczyńska et al. (2021) emphasized that gender also plays an important role, with women experiencing more pain and fatigue during cardiac rehabilitation exercises and demonstrating greater sensitivity, resulting in higher kinesiophobia level. Social support is a key factor, as patients with strong support systems better understand the process and benefits of exercise rehabilitation, reducing fear and improving adherence to rehabilitation programs (Bourke et al., 2022; Li Q. et al., 2024; Liu et al., 2024). Education level also influences kinesiophobia, with higher literacy associated with lower fear of movement (Brunetti et al., 2017; Kluszczyńska et al., 2021; Liu et al., 2024). In addition, Yakut Ozdemir et al. (2023) also found that patients with a regular exercise routine were more likely to maintain physical activity after a cardiac event because they recognized the benefits of moderate exercise and were actively involved in rehabilitation therapy. Low personal monthly income is an independent risk factor for kinesiophobia, as the financial burden of long-term medical costs leads patients to avoid exercise due to concerns about potential physical risks and additional expenses (Shen et al., 2023).

#### 3.5.2 Disease factors

The main disease factors include symptom severity and complications. Patients in the acute phase or with higher cardiac function grades often experience more severe symptoms, such as chest tightness, shortness of breath, dizziness, fatigue, and a sense of impending doom (Zhu et al., 2024). These symptoms heighten their perception of safety threats, leading to increased sensitivity and resistance to physical activity. Conversely, patients in stable phases or with lower cardiac function grades exhibit milder symptoms and lower levels of kinesiophobia (Shen et al., 2023). Demirsoy et al. (2024) found that pain intensity, frequency, and duration correlate with kinesiophobia levels. Pain can amplify stress responses, increasing both pain perception and kinesiophobia (Shen et al., 2023; Liu et al., 2024). Additionally, CHD patients often have comorbidities such as obesity, anemia, hypertension, stroke, diabetes, and heart failure, which may require multiple medications and worsen their kinesiophobia (Bäck et al., 2013; Yakut Ozdemir et al., 2023).

#### 3.5.3 Psychological factors

The primary psychological factors influencing kinesiophobia in CHD patients are anxiety, depression, and self-efficacy. Anxiety, as the main driver, leads to excessive fear of adverse events and avoidance of exercise, while depression, characterized by low mood, fatigue, and negative thoughts, becomes more common after cardiac events due to incomplete recovery and increased risk of future issue (Bäck et al., 2013; Çakal et al., 2022; Wang et al., 2023). Zhang et al. (2023) noted that low exercise self-efficacy, or lack of confidence in performing physical activity, also contributed to avoidance. Positive self-efficacy is linked to good physical and emotional states, while anxiety and fear diminish it (Ren et al., 2022; Song et al., 2022; Wang et al., 2022a).

### 3.6 Consequences

Kinesiophobia in CHD patients is associated with several negative outcomes, including reduced participation and adherence in CR, decreased functional capacity, increased incidence of major adverse cardiovascular events (MACE) and mortality, and lower quality of life (QoL). Kinesiophobia leads to reduced participation and adherence to rehabilitation, with participation rates decreasing by 1% for each day of delayed inclusion (Thomas, 2024). Patients avoid physical activity due to kinesiophobia, which worsens rehabilitation outcome (Bäck et al., 2020; Ren et al., 2022; Yakut Ozdemir et al., 2023). Studies show that higher levels of kinesiophobia correlate with lower physical activity and declining functional capacity, as patients believe exercise may trigger heart issue (Bäck et al., 2017; Khushnood et al., 2023; Ratnoo et al., 2023). This creates a vicious cycle of physical decline, potentially leading to disability (Xiao et al., 2024). Prolonged inactivity increases the risk of MACE and mortality, with early withdrawal from rehabilitation nearly doubling the risk of death or recurrent events (Pardaens et al., 2017). Kinesiophobia also significantly lowers quality of life, as kinesiophobia leads to maladaptation, physical degeneration, and severe psychological distress, further impacting patients’ survival and well-being (Song et al., 2022; Wang et al., 2022b).

### 3.7 Model case

Analysis of typical cases can better help clinical identification, and application of concepts (Tofthagen and Fagerstrøm, 2010). The case described below is a fictionalized account of kinesiophobia in a patient with CHD.

Mr. Wang, a 53-year-old with a history of CHD, hypertension, and heart failure, was admitted for acute myocardial infarction and underwent emergency PCI. Post-surgery, he was advised to remain on bed rest for 24 h. During this time, he frequently worried about his condition, felt ashamed, and viewed himself as weak and unsuccessful, leading to a range of negative emotions such as worry, fear, anxiety, and depression (complex emotional responses). Once stabilized, Mr. Wang was informed he could resume movement, but he resisted, fearing exercise might worsen his symptoms or dislodge his stent, necessitating further surgery (self-symptomatic distress). He also expressed a general reluctance to exercise due to past experiences and a belief that exercise would exacerbate his health issues (subjective avoidance behavior with personality tendencies). Additionally, Mr. Wang admitted to avoiding asking his healthcare provider for guidance on recovery exercises and lacked accurate knowledge about his condition and the benefits of exercise, intensifying his fear of potential negative effects (misperceptions and negative reactions).

### 3.8 Final definition of kinesiophobia in patients with CHD

**Figure 2:**
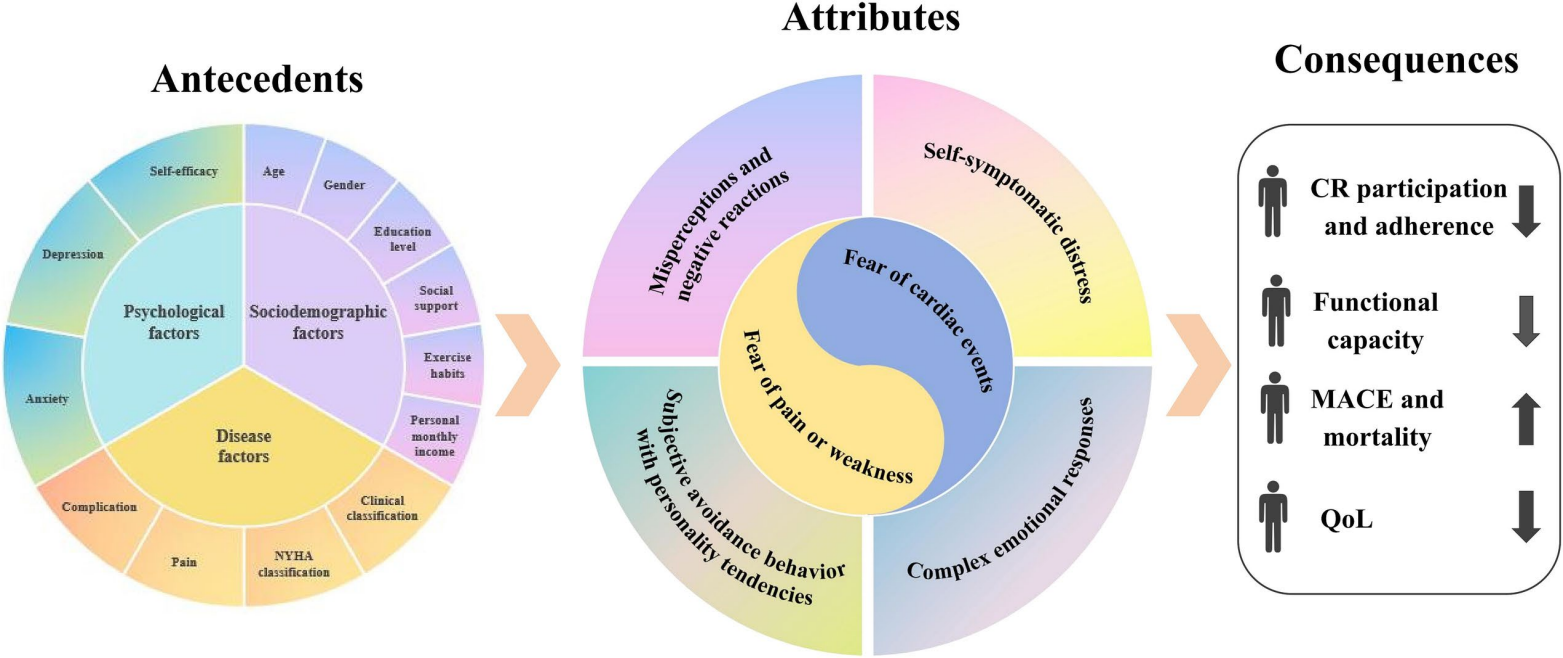
The antecedents, attributes, and consequences of the concept.

Kinesiophobia in patients with CHD is a subjective avoidance behavior that includes both “fear of pain or weakness” and “fear of cardiac events” based on personality tendencies, driven by complex emotional responses and misperceptions based on their own symptomatic disturbances, and presents an excessive and irrational fear of movement. Figure 2 describes the relationship between antecedents, attributes, and consequences.

### 3.9 Measurement tool

Measurement tools define a concept’s characteristics (Tofthagen and Fagerstrøm, 2010). Most assessments of kinesiophobia use general scales rather than specific ones (Jia et al., 2024).

#### 3.9.1 TSK (Tampa scale for kinesiophobia)

The TSK, developed by Kori (1990), is a widely used questionnaire with four dimensions: repetitive strain injury, re-injury, fear avoidance, and work-related injury, totaling 17 items. A higher score indicates greater kinesiophobia, with a Cronbach’s *α* of 0.77. Though translated and used in several countries, the TSK, designed for low back pain, has not been tested for reliability and validity in CHD patients (Roelofs et al., 2011).

#### 3.9.2 TSK-SV heart (Tampa scale for kinesiophobia heart)

The TSK-SV Heart, adapted from the TSK by Bäck et al. (2013), includes four dimensions: danger perception, fear of movement, movement avoidance, and dysfunction, with 17 items. Scores range from 17 to 68, with higher scores indicating greater kinesiophobia. It has a Cronbach’s *α* of 0.78 and has been translated into multiple languages. While the TSK-SV Heart shows strong psychometric properties in Western countries and China, it is intended for a range of heart conditions, not specifically CHD (Ghisi et al., 2017).

#### 3.9.3 Fact-CHD (fear of activity in patients with coronary heart disease)

Developed by Ozyemisci-Taskiran et al. (2020), Fact-CHD is the first tool specifically for measuring kinesiophobia in CHD patients. It assesses individuals who have had a myocardial infarction, coronary artery bypass surgery, or percutaneous coronary intervention in the past 12 months. The 21-item scale has a Cronbach’s α of 0.92, with higher scores indicating greater exercise fear. However, its adoption outside of its initial setting is limited, and further validation is needed for broader applicability.

## 4 Discussion

Clear conceptual frameworks are crucial for advancing nursing knowledge (Tofthagen and Fagerstrøm, 2010). In previous research, kinesiophobia is generally defined as a psychological state where chronic pain patients fear movement due to pain (Kori, 1990). Currently, research on kinesiophobia in CHD patients has mainly addressed prevalence and influencing factors, with less focus on its underlying attributes and mechanisms (Wang et al., 2023; Yakut Ozdemir et al., 2023; Zhu et al., 2024). This article is the first to specifically describe unique characteristics of kinesiophobia in CHD patients, aiming to differentiate it from similar concepts like “exercise anxiety” and other diseases. We concluded that kinesiophobia in CHD is a subjective avoidance behavior driven by personality traits, complex emotional reactions, and misperceptions related to symptoms, resulting in excessive and irrational fear of movement. Unlike other diseases, kinesiophobia in CHD has two dimensions: “fear of pain or weakness” and “fear of cardiac events,” with the latter being more prevalent (Bäck et al., 2013; Shen et al., 2023). Adverse events, such as chest tightness and pain, exacerbate this fear, causing patients to avoid physical activity due to the fear of triggering similar discomfort (Çakal et al., 2022; Yakut Ozdemir et al., 2023). While “exercise anxiety” is a relevant term, it only represents one emotional component of kinesiophobia (Çakal et al., 2022; Liu et al., 2024). Anxiety involves a tendency to act, driven by the desire for success, whereas fear involves avoidance and a flight response. Understanding these distinctions can help healthcare professionals identify kinesiophobia in CHD patients early and develop targeted interventions.

This article defines kinesiophobia in CHD patients across four dimensions: physiological, psychological, cognitive, and behavioral. Key attributes include self-symptomatic distress, complex emotional responses, subjective avoidance behavior with personality tendencies, misperceptions and negative reactions. CHD patients are highly sensitive to bodily signals, with exercise-related sensations often resembling heart attack symptoms, leading to excessive fear (Bäck et al., 2017; Guo et al., 2022; Demirci et al., 2023). Patients’ uncertainty and fear of cardiac tolerance leads to excessive concern about adverse cardiac events (Knapik et al., 2019; Li L. et al., 2024). Therefore, the most important thing for patients with CHD is to strengthen the control and management of disease symptoms. The most direct and effective way to do this is through pharmacologic intervention and interventional therapy, but it is also critical to enhance patient education and awareness of the disease (Shen et al., 2023). Kinesiophobia in CHD patients reflects both a state and a trait, driven by personality and misconceptions about exercise (Wang et al., 2023; Zhu et al., 2024). Misconceptions and outdated beliefs about exercise lead to avoidance and negative attitudes (Demirci et al., 2023; Zhu et al., 2024). Improving patient education through videos, WeChat groups, and articles can enhance understanding and reduce fear (Piao et al., 2022; Yakut Ozdemir et al., 2023). Encouraging peer support and tailored exercise plans, along with professional guidance, can help patients overcome initial fears and promote a positive view of exercise (Bäck et al., 2020).

kinesiophobia in CHD patients is influenced by socio-demographic, disease-related, and psychological factors, leading to reduced participation in cardiac rehabilitation, lower functional capacity, higher rates of cardiovascular events and mortality, and decreased quality of life (Çakal et al., 2022; Yakut Ozdemir et al., 2023). Early identification of risk factors and targeted prevention are essential. Specific tools can identify high-risk patients, but specific scales for assessing kinesiophobia in CHD are scarce (Ozyemisci-Taskiran et al., 2020). Current scales also underexplore the behavioral aspects of kinesiophobia, which involve complex physiological, psychological, cognitive, and behavioral factors. Future research should incorporate a “behavioral dimension” to improve these scales. Moreover, as existing tools rely on subjective self-reports, objective scales from healthcare professionals’ perspectives are needed for greater accuracy. Recent studies have developed validated tools to assess self-management behavior in home-based cardiac rehabilitation, emphasizing the need for structured and comprehensive evaluation methods in cardiac patients (Yang et al., 2023c; Yang et al., 2024a). While some risk prediction tools identify high-risk patients with subacromial pain syndrome (SAPS) and chronic back pain, no such models exist for CHD patients (Panken et al., 2020; Karartı et al., 2023). This reflects a lack of awareness among healthcare professionals regarding early screening for kinesiophobia in CHD, likely due to the late start of research in this area, an unclear definition, and limited exploration of its mechanisms (Bäck et al., 2012). In the future, healthcare professionals must enhance their risk awareness, and consider developing a precise, targeted risk prediction tool using machine learning algorithms to identify high-risk CHD patients with kinesiophobia early, enabling timely, tailored interventions.

Kinesiophobia in CHD patients primarily stems from symptom distress and misperception. After heart-related events, patients become more sensitive to life-threatening situations and develop a heightened fear of death (Bäck et al., 2020; Shen et al., 2023). Cognitive biases make it difficult for them to distinguish between disease-related symptoms and those triggered by exercise, leading them to avoid physical activity out of fear (Bäck et al., 2013; Piao et al., 2022). Early recognition of symptoms, timely psychological intervention, and safe exercise guidance are essential to reducing kinesiophobia. Cognitive-behavioral therapy (CBT) has shown potential in addressing kinesiophobia (Ploutarchou et al., 2023). Guo et al. (2022) applied CBT in 49 patients post-myocardial infarction and found improvements in daily activity performance, reduced kinesiophobia, and alleviated anxiety and depression. However, some studies suggest that CBT may not have short-term effects on kinesiophobia, indicating a need for further investigation (Vergeld et al., 2021). Moreover, as highlighted by Yang et al. (2023b), it is essential to appropriately monitor both objective indicators and subjective status during patients’ physical activity to ensure exercise safety, while also providing essential technical support and education. Additionally, multidisciplinary team rehabilitation may be a promising approach, though it has not been studied in CHD patients. Its effectiveness has been demonstrated in chronic pain management (Monticone et al., 2014). A proposed team could include doctors, nurses, psychologists, and rehabilitation therapists, providing personalized interventions based on patient needs. In this context, establishing an effective social support network becomes a crucial strategy to enhance exercise participation among patients with chronic heart disease (Yang et al., 2024b). It can play a significant role in alleviating kinesiophobia and promoting long-term rehabilitation.

In conclusion, this study conducts a conceptual analysis of kinesiophobia in CHD patients, exploring its core characteristics and unique attributes to provide a clearer theoretical framework for clinical practice. Our findings emphasize that kinesiophobia is a subjective avoidance behavior influenced by individual traits, emotional responses, and cognitive distortions, beyond being merely a psychological condition. This understanding highlights the need for refined assessment tools and the development of targeted interventions. Integrating psychological support, cognitive-behavioral therapy, and multidisciplinary rehabilitation may improve patient outcomes, while structured patient education can correct misconceptions, promote safe exercise participation, and reduce avoidance behaviors. By addressing kinesiophobia comprehensively, healthcare providers can optimize rehabilitation strategies, enhance adherence to exercise programs, and improve long-term cardiovascular health.

### 4.1 Limitations

This study has several limitations. As a concept analysis, it primarily relies on existing literature, which may result in incomplete information. Additionally, our systematic literature search was limited to publications in English and Chinese, potentially overlooking relevant studies in other languages. Furthermore, the analysis was influenced by the availability of existing research and our search strategy. Since this study is not a systematic review, we did not assess the quality of the included studies, which may introduce some bias. However, we adhered to standard practices for systematic evaluation to ensure the rigor of our analysis.

## 5 Conclusion

As research on kinesiophobia in CHD advances, there remains insufficient evidence on its conceptual content. This study analyzed the attributes, antecedents, and consequences of kinesiophobia in these patients, identifying its unique characteristics. Kinesiophobia in CHD patients is a subjective avoidance behavior that includes both “fear of pain or weakness” and “fear of cardiac events” based on personality tendencies, driven by complex emotional responses and misperceptions based on their own symptomatic disturbances, and presents an excessive and irrational fear of movement. This analysis supports the development of specific assessment tools and helps healthcare professionals design individualized intervention plans for patients.
