# Supplementary material for: Kinesiophobia in patients with coronary heart disease: a Rodgers’ evolutionary concept analysis
Source: Front Psychol. 2025 Mar 26;16:1499962. doi: 10.3389/fpsyg.2025.1499962 (PMC11978840; doi:10.3389/fpsyg.2025.1499962)
Supplement: Supplementary file 1 [file Table_1.DOCX]

**Supplementary Materials**

Contents

Supplementary Material 1 Search Strategy Example

Supplementary Material 2 Reasons for final exclusion 54 studies

Supplementary Material 1: Search Strategy Example

**PubMed：**

(("Coronary Disease"[Mesh]) OR ((((((((((((((((((((((Coronary Diseases[Title/Abstract]) OR (Disease, Coronary[Title/Abstract])) OR (Diseases, Coronary[Title/Abstract])) OR (Coronary Heart Disease[Title/Abstract])) OR (Coronary Heart Diseases[Title/Abstract])) OR (Disease, Coronary Heart[Title/Abstract])) OR (Diseases, Coronary Heart[Title/Abstract])) undefined (Heart Disease, Coronary[Title/Abstract])) OR (Heart Diseases, Coronary[Title/Abstract])) OR (coronary artery disease[Title/Abstract])) OR (coronary atherosclerotic heart disease[Title/Abstract])) OR (coronary atherosclerosis[Title/Abstract])) undefined (atherosclerotic cardiovascular disease[Title/Abstract])) OR (Ischemic heart disease[Title/Abstract])) undefined (myocardial ischemia[Title/Abstract])) OR (acute coronary syndrome[Title/Abstract])) OR (myocardial infarction[Title/Abstract])) OR (angina pectoris[Title/Abstract])) OR (percutaneous coronary intervention[Title/Abstract])) undefined (PCI[Title/Abstract])) undefined (Percutaneous transluminal coronary angioplasty[Title/Abstract])) OR (coronary artery bypass[Title/Abstract]))) AND (("Kinesiophobia"[Mesh]) OR ((((((((((((((Pain-Related Activity Avoidance[Title/Abstract]) OR (Activity Avoidance, Pain-Related[Title/Abstract])) OR (Avoidance, Pain-Related Activity[Title/Abstract])) undefined (Pain Related Activity Avoidance[Title/Abstract])) OR (Movement Phobia[Title/Abstract])) undefined (Fear of Movement[Title/Abstract])) OR (Movement Fear[Title/Abstract])) OR (Kinesophobia[Title/Abstract])) OR (Kinetophobia[Title/Abstract])) undefined (Phobia, Movement[Title/Abstract])) OR (fear of reinjury[Title/Abstract])) OR (motion phobia[Title/Abstract])) undefined (fear of activity[Title/Abstract])) OR (fear avoidance[Title/Abstract])))

**Web of Science：**

| **Search Level** | **Search Terms** |  |
| --- | --- | --- |
| Search #1 | TS=(Coronary Disease or Coronary Diseases or Disease, Coronary or Diseases, Coronary or Coronary Heart Disease or Coronary Heart Diseases or Disease, Coronary Heart or Diseases, Coronary Heart or Heart Disease, Coronary or Heart Diseases, Coronary or coronary artery disease or coronary atherosclerotic heart disease or coronary atherosclerosis or atherosclerotic cardiovascular disease or Ischemic heart disease or myocardial ischemia or acute coronary syndrome or myocardial infarction or angina pectoris or percutaneous coronary intervention or PCI or Percutaneous transluminal coronary angioplasty or coronary artery bypass) | [1,574,412](https://www.webofscience.com/wos/alldb/summary/90a0cd40-0cf8-44fe-bcf9-006c520fa926-01018dd4f3/relevance/1) |
| Search #2 | TS=(Kinesiophobia or Pain-Related Activity Avoidance or Activity Avoidance, Pain-Related or Avoidance, Pain-Related Activity or Pain Related Activity Avoidance or Movement Phobia or Fear of Movement or Movement Fear or Kinesophobia or Kinetophobia or Phobia, Movement or fear of reinjury or motion phobia or fear of activity or fear avoidance) | [69,299](https://www.webofscience.com/wos/alldb/summary/e4d5394b-b474-45dc-912a-12712635c6a7-01018ddb0e/relevance/1) |
| Search #3 | #1 AND #2 | [580](https://www.webofscience.com/wos/alldb/summary/644a7521-1cec-4e3d-9900-2b2bd0f2fe27-01018de002/relevance/1) |

**PsycINFO：**

| **Level** | **Search manager** |  |
| --- | --- | --- |
| #3 | #1 AND #2 | 90 |
| #2 | Kinesiophobia OR fear avoidance OR motion phobia OR fear of activity OR Pain Related Activity Avoidance OR Movement Phobia OR Fear of Movement OR Movement Fear OR Kinesophobia OR Kinetophobia OR Phobia, Movement OR fear of reinjury | 14557 |
| #1 | Coronary Disease OR coronary atherosclerosis OR atherosclerotic cardiovascular disease OR Ischemic heart disease OR Coronary Heart Disease OR myocardial ischemia OR acute coronary syndrome OR myocardial infarction OR angina pectoris OR Heart Diseases, Coronary OR coronary artery disease OR coronary atherosclerotic heart disease OR percutaneous coronary intervention OR PCI OR Percutaneous transluminal coronary angioplasty OR coronary artery bypass | 363955 |

**CINAHL：**

| **Level** | **Search manager** |  |
| --- | --- | --- |
| S3 | S1 AND S2 | 11 |
| S2 | Kinesiophobia OR fear avoidance OR motion phobia OR fear of activity OR Pain Related Activity Avoidance OR Movement Phobia OR Fear of Movement OR Movement Fear OR Kinesophobia OR Kinetophobia OR Phobia, Movement OR fear of reinjury | 2599 |
| S1 | Coronary Disease OR coronary atherosclerosis OR atherosclerotic cardiovascular disease OR Ischemic heart disease OR Coronary Heart Disease OR myocardial ischemia OR acute coronary syndrome OR myocardial infarction OR angina pectoris OR Heart Diseases, Coronary OR coronary artery disease OR coronary atherosclerotic heart disease OR percutaneous coronary intervention OR PCI OR Percutaneous transluminal coronary angioplasty OR coronary artery bypass | 37931 |

**the Cochrane Library:**

| **Level** | **Search manager** |  |
| --- | --- | --- |
| #1 | MeSH descriptor: [Coronary Disease] explode all trees | 19047 |
| #2 | (Coronary Diseases):ti,ab,kw OR (Disease, Coronary):ti,ab,kw OR (Diseases, Coronary):ti,ab,kw OR (Coronary Heart Disease):ti,ab,kw OR (Coronary Heart Diseases):ti,ab,kw OR (Disease, Coronary Heart):ti,ab,kw OR (Diseases, Coronary Heart):ti,ab,kw OR (Heart Disease, Coronary):ti,ab,kw OR (Heart Diseases, Coronary):ti,ab,kw OR (coronary artery disease):ti,ab,kw OR (coronary atherosclerotic heart disease):ti,ab,kw OR (coronary atherosclerosis):ti,ab,kw OR (atherosclerotic cardiovascular disease):ti,ab,kw OR (Ischemic heart disease):ti,ab,kw OR (myocardial ischemia):ti,ab,kw OR (acute coronary syndrome):ti,ab,kw OR (myocardial infarction):ti,ab,kw OR (angina pectoris):ti,ab,kw OR (percutaneous coronary intervention):ti,ab,kw OR (PCI):ti,ab,kw OR (Percutaneous transluminal coronary angioplasty):ti,ab,kw OR (coronary artery bypass):ti,ab,kw | 90260 |
| #3 | #1 OR #2 | 90570 |
| #4 | MeSH descriptor: [Kinesiophobia] explode all trees | 33 |
| #5 | (Pain-Related Activity Avoidance):ti,ab,kw OR (Activity Avoidance, Pain-Related):ti,ab,kw OR (Avoidance, Pain-Related Activity):ti,ab,kw OR (Pain Related Activity Avoidance):ti,ab,kw OR (Movement Phobia):ti,ab,kw OR (Fear of Movement):ti,ab,kw OR (Movement Fear):ti,ab,kw OR (Kinesophobia):ti,ab,kw OR (Kinetophobia):ti,ab,kw OR (Phobia, Movement):ti,ab,kw OR (fear of reinjury):ti,ab,kw OR (motion phobia):ti,ab,kw OR (fear of activity):ti,ab,kw OR (fear avoidance):ti,ab,kw | 4112 |
| #6 | #4 OR #5 | 4134 |
| #7 | #3 AND #6 | 62 |

**Embase:**

| No. | Query | Results |
| --- | --- | --- |
| #7 | #3 AND #6 | 50 |
| #6 | #4 OR #5 | 4056 |
| #5 | 'Pain-Related Activity Avoidance':ab,ti OR 'Activity Avoidance, Pain-Related':ab,ti OR 'Avoidance, Pain-Related Activity':ab,ti OR 'Pain Related Activity Avoidance':ab,ti OR 'Movement Phobia':ab,ti OR 'Fear of Movement':ab,ti OR 'Movement Fear':ab,ti OR 'Kinesophobia':ab,ti OR 'Kinetophobia':ab,ti OR 'Phobia, Movement':ab,ti OR 'fear of reinjury':ab,ti OR 'motion phobia':ab,ti OR 'fear of activity':ab,ti OR 'fear avoidance':ab,ti | 3508 |
| #4 | 'kinesiophobia'/exp | 773 |
| #3 | #1 OR #2 | 877806 |
| #2 | 'Coronary Disease':ab,ti OR 'Coronary Diseases':ab,ti OR 'Disease, Coronary':ab,ti OR 'Diseases, Coronary':ab,ti OR 'Coronary Heart Disease':ab,ti OR 'Coronary Heart Diseases':ab,ti OR 'Disease, Coronary Heart':ab,ti OR 'Diseases, Coronary Heart':ab,ti OR 'Heart Disease, Coronary':ab,ti OR 'Heart Diseases, Coronary':ab,ti OR 'coronary atherosclerotic heart disease':ab,ti OR 'coronary atherosclerosis':ab,ti OR 'atherosclerotic cardiovascular disease':ab,ti OR 'Ischemic heart disease':ab,ti OR 'myocardial ischemia':ab,ti OR 'acute coronary syndrome':ab,ti OR 'myocardial infarction':ab,ti OR 'angina pectoris':ab,ti OR 'percutaneous coronary intervention':ab,ti OR 'PCI':ab,ti OR 'Percutaneous transluminal coronary angioplasty':ab,ti OR 'coronary artery bypass':ab,ti | 632652 |
| #1 | 'coronary artery disease'/exp | 435613 |

**Scopus:**( ( TITLE-ABS-KEY ( "Coronary Disease" OR "Coronary Diseases" OR "Disease, Coronary" OR "Diseases, Coronary" OR "Coronary Heart Disease" OR "Coronary Heart Diseases" OR "Disease, Coronary Heart" OR "Diseases, Coronary Heart" OR "Heart Disease, Coronary" OR "Heart Diseases, Coronary" OR "coronary artery disease" OR "coronary atherosclerotic heart disease" OR "coronary atherosclerosis" OR "atherosclerotic cardiovascular disease" OR "Ischemic heart disease" OR "myocardial ischemia" OR "acute coronary syndrome" OR "myocardial infarction" OR "angina pectoris" OR "percutaneous coronary intervention" OR "PCI" OR "Percutaneous transluminal coronary angioplasty" OR "coronary artery bypass") ) AND  ( TITLE-ABS-KEY ( "Kinesiophobia" OR "Pain-Related Activity Avoidance" OR "Activity Avoidance, Pain-Related" OR "Avoidance, Pain-Related Activity" OR "Pain Related Activity Avoidance" OR "Movement Phobia" OR "Fear of Movement" OR "Movement Fear" OR "Kinesophobia" OR "Kinetophobia" OR "Phobia, Movement" OR "fear of reinjury" OR "motion phobia" OR "fear of activity" OR "fear avoidance" ) )

**ProQuest:**

| **Level** | **Search manager** |  |
| --- | --- | --- |
| S3 | [[S1] AND [S2]](https://webvpn.fudan.edu.cn/https/77726476706e69737468656265737421e7e056d2372267416b0d9ab8d6562c38/recentsearches.recentsearchtabview.recentsearchesgridview.scrolledrecentsearchlist.checkdbssearchlink:rerunsearch/E878F39FE204D7BPQ/None/$N?site=healthcomplete&t:ac=RecentSearches) | 12 |
| S2 | [AB,TI("Kinesiophobia" OR "Pain-Related Activity Avoidance" OR "Activity Avoidance, Pain-Related" OR "Avoidance, Pain-Related Activity" OR "Pain Related Activity Avoidance" OR "Movement Phobia" OR "Fear of Movement" OR "Movement Fear" OR "Kinesophobia" OR "Kinetophobia" OR "Phobia, Movement" OR "fear of reinjury" OR "motion phobia" OR "fear of activity" OR "fear avoidance")](https://webvpn.fudan.edu.cn/https/77726476706e69737468656265737421e7e056d2372267416b0d9ab8d6562c38/recentsearches.recentsearchtabview.recentsearchesgridview.scrolledrecentsearchlist.checkdbssearchlink:rerunsearch/3AA38E7F4AC4342PQ/None/$N?site=healthcomplete&t:ac=RecentSearches) | 806 |
| S1 | AB,TI("Coronary Disease" OR "Coronary Diseases" OR "Disease, Coronary" OR "Diseases, Coronary" OR "Coronary Heart Disease" OR "Coronary Heart Diseases" OR "Disease, Coronary Heart" OR "Diseases, Coronary Hear" OR "Heart Disease, Coronary" OR "Heart Diseases, Coronary" OR "coronary artery disease" OR "coronary atherosclerotic heart disease" OR "coronary atherosclerosis" OR "atherosclerotic cardiovascular disease" OR "Ischemic heart disease" OR "myocardial ischemia" OR "acute coronary syndrome" OR "myocardial infarction" OR "angina pectoris" OR "percutaneous coronary intervention" OR "PCI" OR "Percutaneous transluminal coronary angioplasty" OR "coronary artery bypass") | 47923 |

**OVID:**

| # | **Search manager** |  |
| --- | --- | --- |
| 1 | (Coronary Disease or Coronary Diseases or Disease, Coronary or Diseases, Coronary or Coronary Heart Disease or Coronary Heart Diseases or Disease, Coronary Heart or Diseases, Coronary Heart or Heart Disease, Coronary or Heart Diseases, Coronary or coronary artery disease or coronary atherosclerotic heart disease or coronary atherosclerosis or atherosclerotic cardiovascular disease or Ischemic heart disease or myocardial ischemia or acute coronary syndrome or myocardial infarction or angina pectoris or percutaneous coronary intervention or PCI or Percutaneous transluminal coronary angioplasty or coronary artery bypass).ti,ab,kw. | 491729 |
| 2 | (Kinesiophobia or Pain-Related Activity Avoidance or Activity Avoidance, Pain-Related or Avoidance, Pain-Related Activity or Pain Related Activity Avoidance or Movement Phobia or Fear of Movement or Movement Fear or Kinesophobia or Kinetophobia or Phobia, Movement or fear of reinjury or motion phobia or fear of activity or fear avoidance).ti,ab,kw. | 3952 |
| 3 | 1 and 2 | 40 |

**China National Knowledge Infrastructure:**

SU=('冠心病' + '冠状动脉疾病' + '冠状动脉粥样硬化性心脏病' + '冠状动脉粥样硬化' + '动脉粥样硬化性心血管疾病' + '缺血性心脏病' + '急性冠状动脉综合征' + '心肌梗死' + '心绞痛' + '心肌缺血' + '经皮冠状动脉介入治疗' + 'PCI' + '经皮腔内冠状动脉血管成形术' + '冠状动脉搭桥术') AND SU= ('运动恐惧' + '恐动症' + '运动恐惧症' + '害怕运动' + '害怕受伤' + '恐惧回避')

**Wanfang Data:**

题名或关键词:("冠心病" or "冠状动脉疾病" or "冠状动脉粥样硬化性心脏病" or "冠状动脉粥样硬化" or "动脉粥样硬化性心血管疾病" or "缺血性心脏病" or "急性冠状动脉综合征" or "心肌梗死" or "心绞痛"or "心肌缺血" or "经皮冠状动脉介入治疗" or "PCI" or "经皮腔内冠状动脉血管成形术"or "冠状动脉搭桥术") and 题名或关键词:("运动恐惧" or "恐动症" or "运动恐惧症" or "害怕运动" or "害怕受伤" or "恐惧回避")

**VIP Database:**

[(((题名或关键词=心脏康复 OR 题名或关键词=心血管康复) OR 题名或关键词=心脏康复治疗) AND (((((((((((题名或关键词=虚拟现实 OR 题名或关键词=VR) OR 题名或关键词=虚拟现实技术) OR 题名或关键词=虚拟) OR 题名或关键词=灵境技术) OR 题名或关键词=虚拟实境) OR 题名或关键词=视频游戏) OR 题名或关键词=虚拟游戏) OR 题名或关键词=运动游戏) OR 题名或关键词=电脑虚拟活动) OR 题名或关键词=教育虚拟现实) OR 题名或关键词=虚拟现实暴露疗法))](https://cwres.ncu.edu.cn/s/com/cqvip/qikan/G.https/Qikan/search/index?LngMySearHistoryIdGuid=b8fef837-7637-4ad9-8a9e-3ce749fe06c0&from=Qikan_Article_History)

**Chinese Biomedical Literature Database:**

| **序号** | **检索表达式** | **结果** |
| --- | --- | --- |
| 5 | (#4) AND (#3) | 66 |
| 4 | "运动恐惧"[常用字段:智能] OR "恐动症"[常用字段:智能] OR "运动恐惧症"[常用字段:智能] OR "害怕运动"[常用字段:智能] OR "害怕受伤"[常用字段:智能] OR "恐惧回避"[常用字段:智能] | 439 |
| 3 | (#2) OR (#1) | [364277](javascript:historyLink('(#3) OR (#2)')) |
| 2 | "冠状动脉疾病"[常用字段:智能] OR "冠状动脉粥样硬化性心脏病"[常用字段:智能] OR "冠状动脉粥样硬化"[常用字段:智能] OR "动脉粥样硬化性心血管疾病"[常用字段:智能] OR "缺血性心脏病"[常用字段:智能] OR "急性冠状动脉综合征"[常用字段:智能] OR "心肌梗死"[常用字段:智能] OR "心绞痛"[常用字段:智能] OR "心肌缺血"[常用字段:智能] OR "经皮冠状动脉介入治疗"[常用字段:智能] OR "PCI"[常用字段:智能] OR "经皮腔内冠状动脉血管成形术"[常用字段:智能] OR "冠状动脉搭桥术"[常用字段:智能] | [364277](javascript:historyLink('(#3) OR (#2)')) |
| 1 | "冠心病"[不加权:扩展] | [147517](javascript:historyLink(') |

Supplementary Material 2: Reasons for final exclusion 54 studies

**Unavailability of full text (n = 8)**

1. Dynamic prediction method for movement fear risk of patient after percutaneous trans luminal coronary intervention operation for coronary heart disease patients, involves calculating occurrence probability of patient panic disorder after PCI operation
2. The Effect of Incorporating Anti-Gravity Treadmill in a Physical Therapy Treatment at Hospitalization Phase on Post CABG Patients on Mobility, Self-Efficacy, Kinesiophobia and Enjoyment
3. Investigation of the Relationship Between Fear of Movement, Physical Performance and Health-Related Quality of Life in Individuals with Coronary Artery Disease
4. Reduction in Fear of Physical Activity Among Older Persons With Coronary Artery Disease by Physical Therapist–Assisted Exercise: A Randomized Trial
5. Prevalence and the Associated Factors of Kinesiophobia among Patients with Coronary Artery Disease: A Systematic Review and Meta-Analysis
6. Capturing the perspectives of women with coronary artery disease regarding interval training or continuous exercise in cardiac rehabilitation
7. Factors that influence participation in exercise and feelings about health and quality of life in patients following coronary artery bypass surgery.
8. What variables predict participation in exercise-based cardiac rehabilitation in patients with coronary artery disease?

**Topic irrelevance (n = 21)**

1. Spaderna, Modulation of startle and heart rate responses by fear of physical activity in patients with heart failure and in healthy adults
2. Measuring fear of physical activity in patients with heart failure
3. Fear of physical activity relates to cardiac interoception and symptom distress in patients with chronic heart failure
4. The effect of the whole-process care model of the medical union on the improvement of kinesiophobia and bone mineral density in patients with osteoporosis
5. Kinesiophobia and its association with fatigue in CHF patients
6. Kinesiophobia and associated variables in patients with heart failure
7. Fear of physical activity, anxiety, and depression: Barriers to physical activity in outpatients with heart failure?
8. Negative Affect, Type D Personality, Quality of Life, and Dysfunctional Outcomes of Total Knee Arthroplasty
9. A behavioural driving model of adherence to home‐based cardiac rehabilitation exercise among patients with chronic heart failure: A mixed‐methods study
10. Kinesiophobia in patients with cardiovascular disease
11. Kinesiophobia and maladaptive coping strategies prevent improvements in pain catastrophizing following pain neuroscience education in fibromyalgia/chronic fatigue syndrome: An explorative study
12. An investigation of the relationship between fear avoidance beliefs, physical activity and fall risk in male and female patients with chronic disease.
13. Fear of Movement and Low Self-Efficacy Are Important Barriers in Physical Activity after Renal Transplantation
14. Kinesiophobia and its determinants in patients with arteriosclerosis obliterans of the lower limbsImpact of kinesiophobia on initiation of cardiac rehabilitation: A prospective cohort path analysis
15. Fear of movement (kinesiophobia) after cardiac hospitalization: predictors and impact on participation in cardiac rehabilitation
16. The effect of a micro-visual intervention on the accelerated recovery of patients with kinesiophobia after total knee replacement during neo-coronary pneumonia
17. The mediating role of cardiac patients' perception of nursing care on the relationship between kinesiophobia, anxiety and depression in rural hospitals: a cross-sectional study
18. Patients' experiences of a digital group education targeting kinesiophobia after myocardial infarction or atrial fibrillation - A qualitative pilot study
19. The longitudinal relationship between fear of movement and physical activity after cardiac hospitalization: A cross lagged panel model
20. A tailored exposure intervention targeting exercise anxiety and avoidance in cardiac rehabilitation

**Non-English/Chinese language (n = 3)**

1. Change of Life in the Middle age and Elderly Patients with Ischemic Heart Disease (IHD) Relapse after Cardiovascular Interventions
2. مقایسه اثربخشی درمان‌های رفتار درمانی دیالکتیکی و شفقت درمانی بر اضطراب، تحمل آشفتگی و تنظیم هیجانی در بازماندگان بیماری کووید- 19
3. تأثیر آموزش براساس تئوری انگیزش محافظت بر رفتارهای پیشگیریکننده از ابتلا به در دانشآموزان دبیرستانی شهر قم ،A آنفلوانزای نوع

**Registered reports (n = 4)**

1. The effect of physical activity in patients with coronary heart disease using exercise-based rehabilitation application
2. Effects of guided imagery and music in alleviating kinesophobia in patients with coronary heart disease
3. Assessing the effect of preoperative rehabilitation (prehab) program on physical function, balance and fear of fall of patients candidate for coronary artery bypass graft surgery
4. Clinical Pilates (CP) Exercises and Kinesiophobia in CABG

**Incorrect focus (n = 18)**

1. Adherence to rehabilitation and home exercise after myocardial infarction: a qualitative study of expectations, barriers and drivers
2. A PARADIGMATIC BEHAVIORAL-MODEL OF HEART-FOCUSED ANXIETY AND NONANGINAL CHEST PAIN
3. Effects of Home-based Remote Cardiac Rehabilitation on Left Ventricular Function and Fear of Exercise in Patients after Percutaneous Coronary Intervention (PCI): A Retrospective Cohort Study
4. Heart-focused anxiety: An evolutionary concept analysis
5. The role of cardioprotective avoidance beliefs in noncardiac chest pain and associated emergency department utilization
6. The relationship between anxiety sensitivity and clinical outcomes in cardiac rehabilitation: A scoping review
7. REMOTELY DELIVERED MINDFULNESS-BASED COGNITIVE THERAPY FOR SCAD SURVIVORS: FINDINGS OF AN OPEN PILOT TRIAL
8. Columbia Roybal Center for Fearless Behavior Change
9. Conceptualizing Fear of Progression in Cardiac Patients: Advancing our Understanding of the Psychological Impact of Cardiac Illness
10. Perceptions of delay when afflicted by an acute myocardial infarction during the first wave of the COVID-19 pandemic
11. Australian health professionals' perspectives on discussing sexual activity and intimacy with people who have had a heart attack: a qualitative study
12. Determinants of Sexual Health and Sexual Quality of Life after Cardiovascular Surgeries: An Integrative Review
13. Sexual Function, Anxiety, Depression and Coping After Myocardial Infarction: An Exploratory Study
14. Sexual Health After Acute Myocardial Infarction: The Lived Experience of Women During the First-Year Post Discharge
15. Patients' Experiences of Sexual Activity Following Myocardial Ischemia
16. Sexual quality of life in patients undergoing coronary artery bypass graft surgery
17. Sexual rehabilitation after myocardial infarction and coronary bypass surgery: Why do we not perform our job?
18. Gender impact on the correlation between sexuality and marital relation quality in patients with coronary artery disease
